# Supplementary material for: A Functional Magnetic Resonance Imaging Investigation of Hot and Cool Executive Functions in Reward and Competition
Source: Sensors (Basel). 2025 Jan 29;25(3):806. doi: 10.3390/s25030806 (PMC11820429; doi:10.3390/s25030806)
Supplement: Supplementary file 1 [file sensors-25-00806-s001.zip › sensors-3316328-supplementary.pdf]

A Functional Magnetic Resonance Imaging Investigation of Hot and Cool Executive Functions in Reward and Competition

Hsin-Yu Lin [1], Hoki Fung [2,3], Yifan Wang [4], Roger Chun-Man Ho [5] and Shen-Hsing Annabel Chen [1,2,6,*]

[1]Centre for Research and Development in Learning, Nanyang Technological University, Singapore 637335, Singapore; linhy@ntu.edu.sg
[2]Psychology, School of Social Sciences, Nanyang Technological University, Singapore 639818, Singapore
[3]Department of Psychiatry and Biobehavioral Sciences, Semel Institute for Neuroscience and Human Behavior, University of California, Los Angeles, CA 90095, USA; hokifung@berkeley.edu
[4]Key Laboratory of Modern Teaching Technology, Ministry of Education, Shaanxi Normal University, Xi'an 710062, China; wangyifan0929@126.com
[5]Department of Psychological Medicine, Yong Loo Lin School of Medicine, National University of Singapore, Singapore 119228, Singapore; pcmrhcm@nus.edu.sg
[6]Lee Kong Chian School of Medicine, Nanyang Technological University, Singapore 636921, Singapore
*Correspondence: annabelchen@ntu.edu.sg

Supplementary Materials

Table S1. fMRI analysis contrasts

No.	Competition	Reward
1	No	High reward - Rest
2	No	No reward - Rest
3	No	High reward - No reward
4	Yes	High reward - Rest
5	Yes	No reward - Rest
6	Yes	High reward - No reward
7	No - Yes	High reward - Rest
8	No - Yes	No reward - Rest
9	No - Yes	High reward - No reward
10	Yes - No	High reward - Rest
11	Yes - No	No reward - Rest
12	Yes - No	High reward - No reward

Figure S1. Contrasts referenced in Figure 3

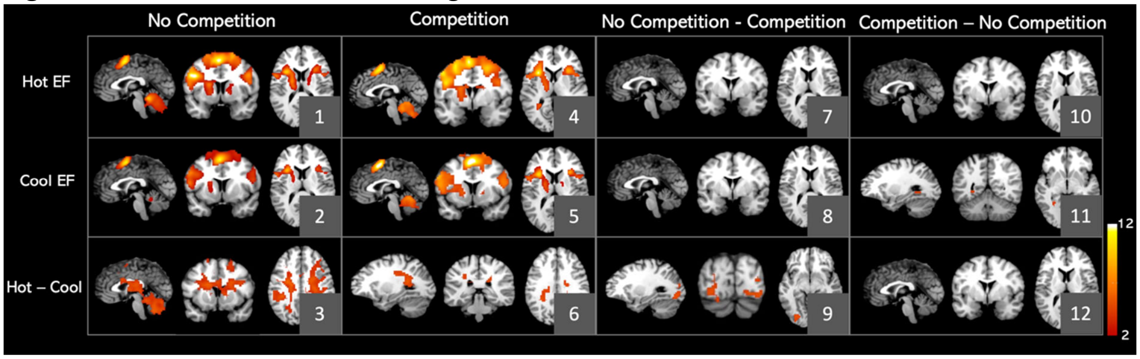

Table S2. Debriefing

Debriefing questions	Subjects (*n* = 29)
Think differently in reward trials	48.28%
Think differently in competition trials	62.7%
Act differently in competition trials	42.86%
Believe the cover story*	68.97%
Importance of winning the money[1]	3.28 (1.22)
Importance of the amount of reward[1]	2.79 (1.26)
How much they like the competitor[2]	3.14 (0.69)
Importance of earning the points in BART[1]	3.90 (0.94)

The variables are demonstrated as mean (standard deviation).
*No significant difference in task performance was found between participants who believed the story and those who did not.
[1]The participants were asked to rate on a scale of 1 to 5 (1= not important at all, 5= extremely important).
[2]The participants were asked to rate on a scale of 1 to 5 (1= not at all, 5= extremely like).

Figure S2. Power analysis results for the fMRI design

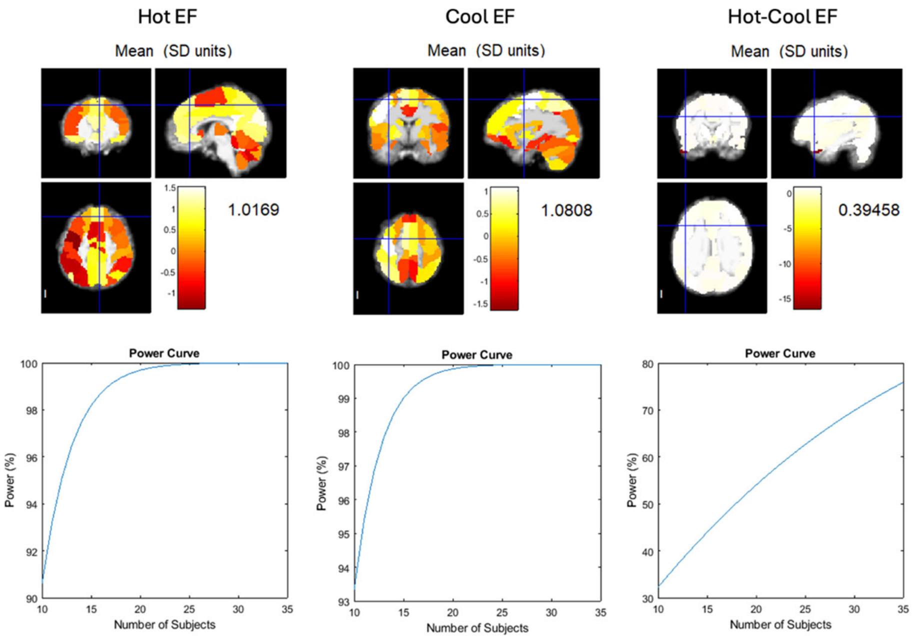

A power analysis was conducted using fmripower (http://fmripower.org/) to estimate the required sample size for detecting significant effects in our fmri design. The analysis, with a Type I error rate of 0.05, indicated that including more than 28 subjects would achieve a desired power range of 70% to 99% for reliably detecting group-level effects.
